# Perspectives of diverse Spanish- and English-speaking patients on the clinical use of polygenic risk scores

**DOI:** 10.1016/j.gim.2022.03.006

**Published:** 2022-04-05

**Authors:** Sabrina A. Suckiel, Giovanna T. Braganza, Karla López Aguiñiga, Jacqueline A. Odgis, Katherine E. Bonini, Eimear E. Kenny, Jada G. Hamilton, Noura S. Abul-Husn

**Affiliations:** 1 The Institute for Genomic Health, Icahn School of Medicine at Mount Sinai, New York, NY;; 2 Department of Medicine, Icahn School of Medicine at Mount Sinai, New York, NY;; 3 Department of Genetics and Genomic Sciences, Icahn School of Medicine at Mount Sinai, New York, NY;; 4 Department of Psychiatry & Behavioral Sciences, Memorial Sloan Kettering Cancer Center, New York, NY;; 5 Department of Medicine, Memorial Sloan Kettering Cancer Center, New York, NY;; 6 Weill Cornell Medical College, New York, NY

**Keywords:** Diverse populations, Genomic medicine, Genomic risk, Polygenic risk scores, Risk communication

## Abstract

**Purpose:**

As polygenic risk scores (PRS) emerge as promising tools to inform clinical care, there is a pressing need for patient-centered evidence to guide their implementation, particularly in diverse populations. Here, we conducted in-depth interviews of diverse Spanish- and English-speaking patients to explore their perspectives on clinical PRS.

**Methods:**

We enrolled 30 biobank participants aged 35–50 years through a purposive sampling strategy, ensuring that >75% self-reported as African/African American or Hispanic/Latinx and half were Spanish-speaking. Semistructured interviews in Spanish or English explored attitudes toward PRS, barriers to adoption, and communication preferences. Data were analyzed using an inductive thematic analysis approach.

**Results:**

Perceived utility of clinical PRS focused on the potential for personal health benefits, and most participants stated that high-risk results would prompt physician consultations and health behavior changes. There was little concern among participants about the limited predictive power of PRS for non-European populations. Barriers to uptake of PRS testing and adoption of PRS-related recommendations included socioeconomic factors, insurance status, race, ethnicity, language, and inadequate understanding of PRS. Participants favored in-person PRS result disclosure by their physician.

**Conclusion:**

Findings provide valuable insight into diverse patients’ attitudes and potential barriers related to clinical PRS, guiding future research and patient-centered clinical implementation.

## Introduction

Recent years have seen increasing opportunities to integrate complex genetic information into risk models for preventive medicine. In particular, polygenic risk scores (PRS), which combine information from hundreds to millions of genetic variants associated with common diseases into a single predictive score, are emerging as promising clinical tools to estimate risks for a range of common diseases.^1^ However, data to support the integration of PRS into clinical care are lacking,^2–4^ and there are important concerns that the use of clinical PRS could deepen health disparities.^5^ Currently, the predictive power of PRS is greater in European descent populations than in others,^5,6^ which may adversely affect clinical utility in underrepresented populations and could influence people’s perceptions of the personal value of PRS. Furthermore, sociodemographic factors, including race and ethnicity, insurance status, and education, can influence access to genomic medicine and testing more generally,^7^ stressing the importance of undertaking equitable approaches to clinical PRS implementation.

The translation of PRS into improved health outcomes relies heavily on patients’ uptake of clinical PRS testing and adoption of PRS-related medical recommendations. Presently, little is known about how patients might perceive and use this emerging technology. A small number of studies exploring people’s reactions to receiving PRS found that many identified personal value in this type of risk information.^8–10^ A more substantial body of literature involves early adopters of other types of preventive genomic testing, including genome sequencing, genotype-based testing, and direct-to-consumer offerings. This research has found that despite interest in obtaining personal genomic risk information,^11–14^ many people do not modify their health behaviors on the basis of this information.^15–18^ In contrast, recent studies have shown that incorporating PRS into clinical risk assessments can lead to uptake of risk-reducing interventions^19,20^ and clinical benefit.^20^ However, the majority of these studies involved predominantly European descent and highly educated individuals, limiting their generalizability.

As polygenic risk information becomes increasingly available,^21,22^ there is an urgent need to develop an evidentiary foundation for clinical PRS implementation in diverse populations. To realize universal benefit, the perspectives of racially and ethnically diverse, multilingual, and medically underserved individuals must be an integral component of this foundation. A major goal of the National Human Genome Research Institute–funded Electronic Medical Records and Genomics (eMERGE) 4 Network is to generate evidence for clinical PRS implementation. Through an ongoing prospective study that will recruit 25,000 patients across 10 US sites, PRS will be generated for 10 common conditions, genomic risk communicated, and outcomes evaluated.^23^ As part of eMERGE 4, this study aimed to evaluate perspectives on clinical PRS among a racially, ethnically, and linguistically diverse patient population in New York City. We interviewed Spanish- and English-speaking patients to generate patient-centered evidence to guide clinical PRS implementation in eMERGE and more broadly.

## Materials and Methods

### Participants

The study included 30 patients of the Mount Sinai Health System who are participants of the Bio*Me* Biobank. Bio*Me* comprises approximately 60,000 participants enrolled from ambulatory settings across the health system, of whom more than 65% self-report as non-European descent.^24^

### Recruitment

Bio*Me* participants indicating Spanish or English as their preferred language and between the ages of 35 and 50 years (to capture healthy individuals as well as individuals with one or more common conditions) were eligible. A stratified purposive sampling strategy was used to ensure that >75% of enrolled participants self-reported as African, African American, or Black (AA) or as Hispanic/Latinx (H/L), and at least half reported Spanish as their preferred language. Research coordinators mailed Spanish and English invitation letters and subsequently followed up by telephone to describe the study, confirm eligibility, and schedule interviews. A total of 30 individuals were enrolled to allow for thematic saturation in both language groups.^25^ Individuals received a $60 gift card for their participation.

### Interview approach

A semistructured interview guide was developed by members of the study team with expertise in medical genetics, genetic counseling, public health, and health psychology. The guide consisted of a uniform set of open-ended questions and discussion prompts to facilitate enriching responses and explored 3 key domains: attitudes toward PRS, barriers to PRS adoption, and preferences for delivery of PRS results (Supplemental Table 1).

Given the limited awareness of PRS among the general population, we presented a 7-minute educational module at the beginning of each interview to familiarize participants with PRS. This prerecorded, narrated PowerPoint presentation explained PRS, how they can be used, and current limitations, including increased accuracy in populations with origins from Europe compared with other populations. A vignette describing a scenario where the participant obtained a PRS for hypercholesterolemia was included to help contextualize using clinical PRS for common health conditions. Results specific to the hypercholesterolemia vignette are not reported here.

Before watching the educational module, participants responded to 2 questions assessing baseline familiarity with genetics that were adapted from previous research^11^ and included (1) rating their understanding of genetics on a 5-point scale from none to high and (2) stating whether they knew the meaning, were aware of, or had never heard of 5 different genetic terms. Participants were also asked if they had ever undergone genetic testing. On concluding the interview, participants completed a survey capturing sociodemographic characteristics.

Interviews were conducted virtually via a secure Zoom platform in Spanish (KLA) or English (GTB, SAS) and were 60–90 minutes long. All interviews were audio-recorded and were transcribed and translated through a Health Insurance Portability and Accountability Act–compliant transcription service.

### Data analysis

Interview data were analyzed using an inductive thematic analysis approach.^26,27^ An initial codebook was developed on the basis of the interview guide and was refined through an iterative process involving discussions among 5 analysts (SAS, GTB, KLA, JAO, KED) and applying iterations of the codebook to a series of 4 transcripts (1 Spanish and 3 English). Each transcript was independently coded by 2 analysts using Dedoose v8.3.45. Any coding discordance was resolved through discussion among the analysts. The analysis team reviewed coded excerpts, identified emergent themes, and determined that thematic saturation was achieved. A subsequent analysis explored whether participants’ perspectives differed by preferred language by stratifying coded excerpts by preferred language before review. Finally, themes and subthemes were defined and categorized by the analysis team.

## Results

A total of 30 participants were interviewed, including 15 who were Spanish-speaking (Table 1). Participants were 50% H/L, 30% AA, 7% White, and 3% Asian by self-report. Overall, 63% were born outside the United States. Participants’ median age was 45 years (range, 35–50), 57% identified as female, 43% had an annual household income <$20,000, and 53% had a high school degree or lower educational level. The cohort included more AA and H/L individuals (who were purposely oversampled), lower income, and similar education levels as New York City population averages.^28^ The majority reported either none/minimal (40%) or some (43%) understanding of genetics. Before watching the educational module, only 2 participants knew the meaning of polygenic risk score.

Emergent themes relating to clinical PRS implementation corresponded to 5 categories: interest, perceived utility, concerns, barriers, and communication preferences. Themes and subthemes are *italicized* throughout the text. Themes, subthemes, and illustrative quotes related to interest appear in Supplemental Table 2, perceived utility in Table 2, concerns in Table 3, and barriers in Table 4. Observed differences based on preferred language are noted in the text.

### Interest

Most participants expressed interest in obtaining clinical PRS, which is similar to previous reports involving preventive genomic testing.^11–14^ Interest stemmed mainly from the potential to *gain health-related knowledge*, *mitigate disease risk*, and *provide information for family members* and was influenced by *family health history* and *trust in medical science*. See Supplemental Table 2 for details on themes and subthemes relating to interest.

### Perceived utility

#### Personal health benefits

The utility of PRS was primarily viewed as the potential for *personal health benefits* (Table 2). Many participants identified value in learning about their personal disease risk and said they would seek physicians’ recommendations and make medical and lifestyle modifications to address their risk. Participants also discussed the possibility for PRS to lead to personalized medical interventions. However, some individuals acknowledged that clinical utility is tied to the availability of established risk-reducing interventions. Participants *prioritized the utility of PRS-related information by disease type* and believed that risk information for certain diseases would be more personally useful than for other diseases. For example, some believed that PRS for heart disease and cancer were of the highest priority, especially if they had limited financial resources available for medical care.

Participants identified factors influencing patients’ ability to act on PRS results, thereby affecting clinical utility, including *personal initiative* and an *adequate understanding of next steps* (eg, medication adherence, lifestyle changes). Many participants claimed that personality, willpower, denial, and personal beliefs would dictate whether individuals adapted their health behaviors to reduce PRS-identified disease risk. Others worried that without proper guidance, they would not know what to do with their results.

Finally, some individuals believed that PRS would provide *no additional gain to personal health,* which mainly stemmed from confidence in their current health status. A few participants suggested that whether or not PRS information was personally useful may depend on a *person’*s *age* because people may be more receptive to addressing disease risk during certain stages in life.

#### Family and community health benefits

A secondary theme related to perceived utility was the potential for *family* and *community health benefits*. Most participants identified value in PRS providing information beyond themselves to their relatives. In addition, some mentioned that a greater understanding of risks associated with diseases prevalent among certain racial and ethnic groups would confer benefits to their community and society at large. For instance, one participant thought that the AA community would benefit from a PRS for diabetes because she recognized a high prevalence of this disease in her community.

### Concerns

#### Psychological implications

The foremost concern raised by participants was the *potential for high-risk results to lead to psychological distress* (Table 3). Almost half of participants thought they would experience *concern, worry, and even fear about receiving a high-risk result*. As if in reaction to the imagined emotional burden of high-risk results, some participants discussed ways to mitigate their disease risk and the availability of preventive treatment. In addition, a small proportion thought that knowledge of health risks alone could lead to disease, described as a self-fulfilling prophecy. *Anticipating disease onset* was another aspect of receiving high-risk PRS results that participants thought would lead to psychological distress because not knowing when or if a disease would present could be anxiety-provoking.

#### Accuracy

Participants expressed a *mix of concerns about the accuracy of PRS*, with close to equal proportions stating that they had or did not have concerns about accuracy. For those who expressed concerns, these were mainly about the possibility for inaccurate results (eg, false positives, false negatives) or low predictive value. Participants without concerns regarding accuracy stated that they trusted in research and science, and some stated that they had no reason to believe PRS testing would be inaccurate.

The educational module included information on the limited predictive accuracy of PRS in non-European descent populations. Nonetheless, there was *little concern about the accuracy of PRS in diverse populations*. Only 2 participants expressed concerns about this limitation, even when explicitly asked by the interviewer. A third participant stated that they would be hesitant to have PRS testing if they were not of European descent. Most participants who commented on this limitation stated that it did not worry them, suggesting little or no impact on interest in PRS testing. One participant said, “For the most part, I guess the more people they test, the more they can compare it to…I still would like to know” (P07, English-speaking). Furthermore, 2 participants who did not express concerns thought that by electing to have a PRS test, they would be contributing to efforts to diversify genomic research and improve the accuracy of PRS for diverse populations. As one participant explained, “I’m Caribbean, so if doing it on me could benefit somebody, then I’m happy to do it. Maybe they can learn something from my genetic trait” (P27, English-speaking).

#### Privacy

A few participants imagined that PRS testing could lead to *loss of privacy and the misuse of an individual’s genetic and/or health information*, including one participant who thought that their information could potentially be sold. However, others stated that they were not concerned about privacy; for instance, a participant stated that their privacy was protected because medical offices must ask permission before sharing information.

### Barriers

#### Access disparities

Participants identified *access disparities* as the main barrier to broad adoption of clinical PRS, encompassing socioeconomic factors that could affect access to PRS testing, availability of post-test medical care, and the ability to follow PRS-related medical recommendations (Table 4). Most participants discussed general difficulties accessing quality health services, with some highlighting that PRS testing may not be offered at every hospital and/or clinic. For example, one participant said a barrier is “a place to get it if you’re in a small town that doesn’t have a very sophisticated hospital or lab” (P17, English-speaking). Cited obstacles to the uptake of PRS-related medical recommendations included lack of access to healthy foods, medications, transportation to appointments, and social support. One participant said “… ability to have food. Sometimes people don’t have money to buy good food and eat right” (P31, Spanish-speaking).

##### Financial barriers

Financial barriers that could limit access to testing and/or uptake of medical recommendations was the most prominent subtheme related to access disparities, discussed by more than three-fourth of participants. One participant spoke of the financial burden of post-test medical care, saying “if you come out with the disease and it requires a lot of money…you are going to worry a lot, but then you got to say so what I’m going to do now? There’s nowhere to find that kind of money” (P18, Spanish-speaking). Furthermore, some participants noted that challenges present among people with limited financial resources (eg, working long hours) might contribute to a reluctance or inability to seek PRS testing and preventive care more broadly.

##### Health insurance coverage

Health insurance coverage was also related to potential disparities in access to clinical PRS. Almost half of participants mentioned that although they may have access to health insurance, they would worry about out-of-pocket costs associated with PRS testing. Additional cited obstacles to obtaining testing included being denied access to genetic testing by insurance, high co-pays, and lack of in-network providers who could order testing.

A few participants noted the *potential for limited access to clinical PRS based on race and ethnicity or preferred language,* discussing inequitable access to health care and genetic testing for diverse populations. Two viewpoints were uniquely expressed by the Spanish-speaking cohort. First, when language discordance between the patient and provider exists, using a language interpreter does not always resolve communication issues. Consequently, patients may not adequately be able to communicate interest in testing, as highlighted by a participant who said “So I don’t ask that question, I just ask something superficially and that’s it. The language is an obstacle” (P35, Spanish-speaking). Second, a few Spanish-speaking participants mentioned that immigrants encounter less access to health insurance coverage and subsequently would have difficulty accessing clinical PRS.

#### Understanding

Almost two-thirds of participants acknowledged *limited understanding of PRS* as a potential obstacle to patients using PRS-based risk information. They conveyed a general lack of confidence in their and others’ ability to understand PRS results, implications of results, and next steps for medical care. One participant thought that patients might completely dismiss their health risks because of inadequate understanding. A small number of individuals cited *limited health and genomic literacy* as a potential contributor to a lack of comprehension.

Participants discussed strategies for improving patients’ understanding of PRS-related information, highlighting *language as a key element to understanding*. Many emphasized that providers should use *nontechnical, lay language* because patients are often not experts in genetics and do not understand genetic terminology. Almost all Spanish-speaking participants highlighted the importance of *communicating in the patient’s own (preferred) language* to help support their understanding of the information. Some also indicated that written materials should be provided in Spanish. Furthermore, a few bilingual participants preferred hearing results in Spanish (their primary language) to ensure their understanding of clinical information.

### Communication of PRS results

**Figure 1:**
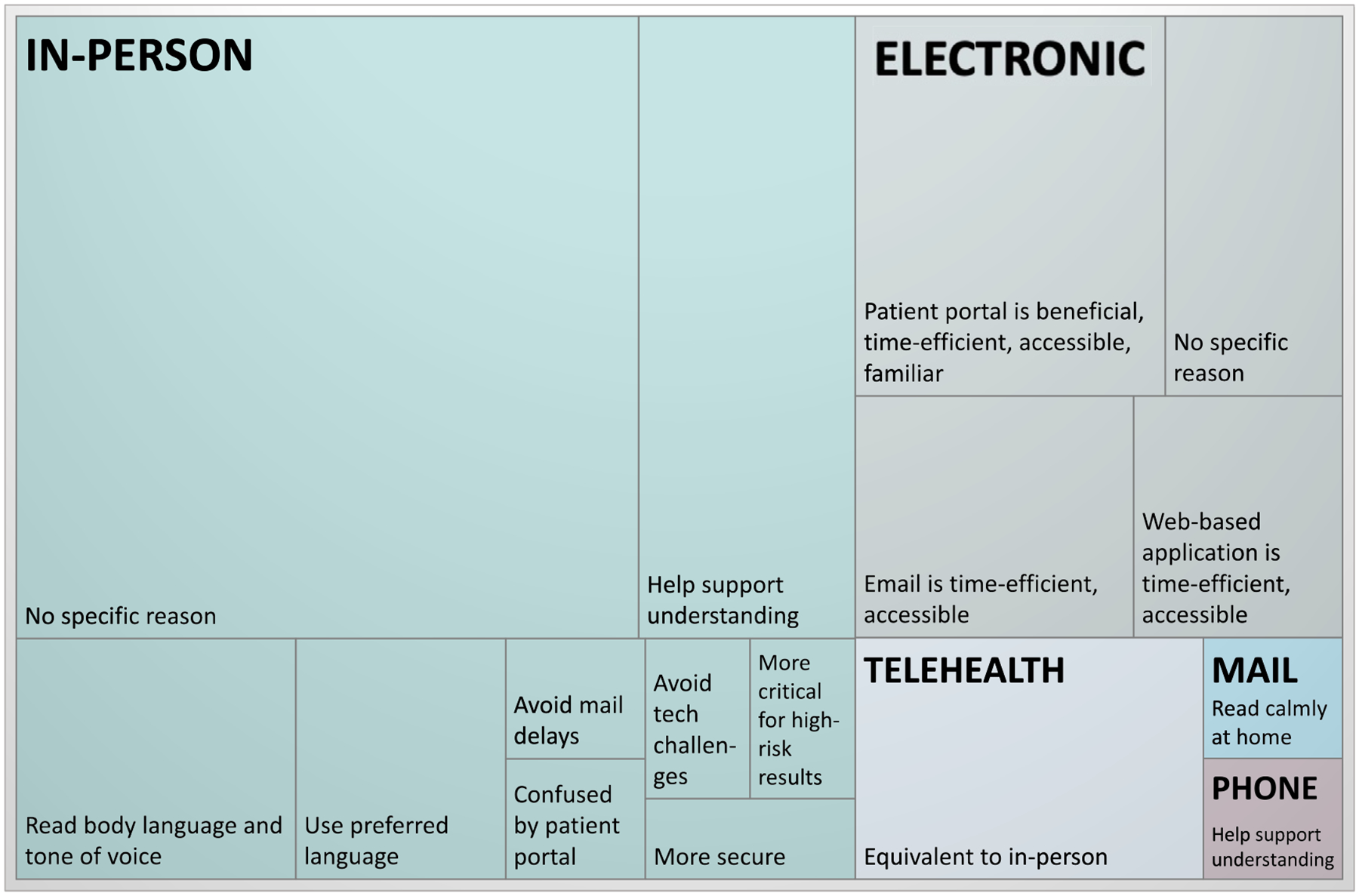
Preferred methods for clinical PRS result disclosure and rationale. The majority of participants favored in-person disclosure, followed by electronic and telehealth delivery. Few participants preferred receiving PRS results by mail or by telephone. To produce the treemap, data corresponding to the best method for result disclosure, a topic included in the interview guide, were coded, categorized, and then tallied to determine the hierarchy. When multiple modalities were described as equally acceptable, all cited modalities were included in the tally. Participants’ rationale for supporting a specific disclosure method was also captured, and similar reasons were grouped and tallied. PRS, polygenic risk score.

The majority of participants preferred *in-person PRS result disclosure* to alternative disclosure methods (eg, electronic, telephone). Participants thought that an in-person appointment would facilitate communication between the patient and provider. In addition, participants valued being able to read their provider’s body language and tone of voice, thereby allowing them to assess the severity or seriousness of the results. One participant explained their preference for in-person disclosure, saying “…there’s nothing like a conversation face-to-face, it’s a different way of communicating; you can actually see the person, their body language, it speaks for itself” (P08, English-speaking). Electronic result disclosure methods (eg, patient portal, mobile health applications, email) were viewed as the second-best option because of their accessibility and time efficiency. However, many participants thought that follow-up discussions with a provider should be available. Figure 1 displays participant preferences and rationale for the preferred method of PRS result disclosure.

Participants stated that their preferred *method of result disclosure depended on the result category*, which, for this study was described as either high risk or not high risk for disease. Almost half of participants thought that PRS results should be returned in person to best support understanding, regardless of result category. Others viewed not high-risk results as less crucial and eligible for being returned electronically or by mail.

Most participants’ initial reaction to being asked who should disclose clinical PRS results was “my physician” (ie, their primary care provider). A few stated that they would be satisfied having a nurse practitioner return PRS results. Almost all participants were previously unaware of genetics specialists; however, when prompted, many were receptive to receiving results through a medical geneticist or genetic counselor.

## Discussion

The clinical application of PRS could broadly affect precision medicine, but there is a paucity of patient-centered data on integrating this genomic innovation into clinical care so that all populations benefit. To this end, we conducted 30 interviews in Spanish and English to explore patients’ views on clinical PRS. The study cohort comprised participants generally underrepresented in genomic research,^7,29,30^ because the majority were AA or H/L, half were Spanish-speaking, and more than half were born outside the United States. We found that although interest in and perceived value of clinical PRS was high among most study participants, identified barriers affecting diverse communities could hinder equitable access and widespread adoption of PRS.

The potential for clinical PRS to confer personal health benefits was central to how participants perceived the utility of testing and aligns with the anticipated clinical utility of PRS-based risk prediction.^4,31^ Most individuals stated that they would seek their physician’s advice and change their health behaviors based on a PRS result indicating high disease risk, which corresponds to findings from a study investigating the clinical utility of PRS in cardiovascular disease.^20^ At the same time, participants considered the utility of clinical PRS within the context of limited resources, weighing the value of disease risk information against the financial burden. This suggests that the tension between interest in PRS and the need to allocate personal resources may be an important factor in patient decision making. Exploration of clinical PRS-based patient decision making, particularly in the context of complex cultural and health access factors, may help develop tailored patient decision support strategies.

Concerns exist owing to the reduced predictive value of PRS in diverse and multiethnic populations and the subsequent potential for clinical PRS implementation to deepen existing health disparities.^5,6^ In this study, only a few participants expressed unease about the limited accuracy of PRS results in underrepresented populations, even though this was highlighted in the educational module reviewed before the interview. It is possible that the educational module did not convey enough information for participants to fully appreciate this limitation. Alternatively, participants may have been willing to overlook this limitation given the potential benefits of PRS to themselves or to others in their communities in the future. Although research efforts to improve the transferability of PRS across populations are ongoing,^23,32,33^ simultaneous efforts are needed to gain a thorough understanding of patient perspectives on this topic and to develop strategies to effectively and transparently communicate PRS limitations that enable patients to make informed testing decisions.

Another important theme that emerged was that access to clinical PRS could be limited in diverse populations. Disparities in genetic testing based on race and ethnicity persist,^34,35^ and this finding amplifies concerns previously raised about the equitable implementation of PRS in diverse populations.^5^ Participants identified socioeconomic barriers to clinical PRS uptake and adoption of PRS-based recommendations, including limited health care access, inadequate insurance coverage, transportation challenges, and insufficient financial means. These perspectives underscore the importance of considering barriers to initial uptake of genomic testing innovations, as well as barriers that patients and communities may face to adopt risk-reducing behavioral and medical strategies. Including populations of diverse racial and ethnic backgrounds and socioeconomic status in PRS implementation research is critical to defining, and ultimately resolving, these challenges, which has been emphasized for genomic medicine more broadly.^36^

An additional cited barrier to using PRS was an inadequate understanding of the results. Most study participants had minimal genetic awareness and lower educational attainment, and half encountered communication challenges when there was a language discordance with their provider. Spanish-speaking study participants expressed the need for direct communication with health care providers in their preferred language to support their understanding. Given that genomic risk information is inherently complex and that there is limited health literacy and English-language proficiency among US adults,^37,38^ thoughtful consideration of how PRS results are communicated is critical. For example, although participants discussed limited genomic literacy as a potential barrier to understanding, PRS may eventually be used analogously to nongenetic clinical risk information (eg, as variables incorporated into clinical risk calculators), in which case patients may not need ample genomic literacy, per se, to interpret their disease risk accurately. Approaches to delivering PRS results are evolving, including recently proposed design strategies for clinical PRS reports that may improve patient engagement.^39,40^

Finally, the mode of result delivery may also influence patient understanding and adaptation to their genomic risk. Participants preferred in-person result disclosure with their primary care physician. These findings suggest that clinical and research programs, such as the eMERGE Network, would benefit from involving multilingual clinicians in returning PRS results; developing patient-friendly, multilingual educational and decision support materials; and eliciting patient/participant preferences for the method of result return.

This study allowed for the in-depth assessment of perspectives from patients who are diverse with regard to race and ethnicity, language, socioeconomic status, and US nativity, backgrounds that are inadequately represented in genomic medicine research. Nonetheless, there are limitations to the study. Because of the COVID-19 pandemic, interviews were conducted virtually, which may have allowed for more distractions compared with in-person interviews. We enrolled participants aged 35–50 years, which excluded the perspectives of children and adults of other ages who may also use clinical PRS. All study participants had previously participated in a research biobank at Mount Sinai, potentially indicating heightened receptivity to genomics research compared with the general population. Differences in themes were analyzed by preferred language, but language could be a proxy for other important factors, such as country of origin, acculturation level, or immigration status. Finally, elicited patient views on PRS were based on hypothetical offerings, which may differ from the actual uptake and adoption of clinical PRS.

## Conclusion

We explored the perspectives of diverse, Spanish- and English-speaking patients on the clinical use of PRS. Patients expressed enthusiasm for clinical PRS, identified the potential for PRS to inform preventive care, and were motivated to address PRS-informed health risks. Importantly, patients highlighted the possibility for PRS results to cause psychological distress. They also identified several potential barriers to clinical PRS uptake, including access disparities, health insurance coverage, and limited understanding of PRS-related information. These findings highlight important patient-centered considerations for current and future PRS implementation in research and clinical care.

## Supplementary Material

Supplementary Material

## Figures and Tables

**Table 1:** Participant characteristics (*N* = 30)

Characteristic	N (%) or Median (Range)
Age	45 (35–50)
Gender identity	
Male	12 (40)
Female	17 (57)
Nonbinary	1 (3)
Self-reported race/ethnicity	
Asian	1 (3)
AA	9 (30)
H/L	15 (50)
White	2 (7)
Multiple selected	2 (7)
Prefer not to answer	1 (3)
Country of origin	
United States	11 (37)
Outside the United States Chile (1), Cuba (1), Dominica (1), Dominican Republic (2), Ecuador (1), El Salvador (2), Lesotho (1), Mexico (5), Paraguay (1), Puerto Rico (3), South Korea (1)	19 (63)
Preferred language	
English	15 (50)
Spanish	15 (50)
Annual household income	
Less than $20,000	13 (43)
$20,000 to $39,999	5 (17)
$40,000 to $59,999	5 (17)
$60,000 to $79,999	4 (13)
Greater than $80,000	3 (10)
Highest level of education completed	
Less than high school	3 (10)
Some high school	4 (13)
High school graduate	9 (30)
Some post-high school training	9 (30)
Bachelor’s degree	3 (10)
Graduate or professional degree	2 (7)
Previous genetic testing	7 (23)
Self-rated understanding of genetics	
None/minimal	12 (40)
Some	13 (43)
Moderate/high	5 (17)
Know the meaning of the genetic term	
DNA	17 (57)
Gene	11 (37)
Genetic testing	10 (33)
Genetic/genomic risk	8 (27)
Polygenic risk score	2 (7)

*AA*, Black, African American, or African; *H/L*, Hispanic/Latino.

**Table 2:** Perceived use of clinical PRS

Themes/Subthemes	Example Quotes
**Personal health benefits**	*“I’m already grabbing the bull by the horns and I’d say, if I’m already aware of this I’m going to start taking preventive measures, that I’ll do this and that and start with my home remedies.” (P31, Sp-speaking)*
Prioritized the utility of PRS-related information by disease type	*“It’s good, for example, for a person that doesn’t have cholesterol that test would be very useful, but for someone like me, who already has it, we wouldn’t understand if this result would be good or bad.” (P39, Sp-speaking)*
Personal initiative	*“No, it depends on the person and their attitude, and how much the person love life, how much the person knows about their body and love themselves and their body. It depends on a person.” (P10, Eng-speaking)*
Adequate understanding of next steps	*“Well not knowing what steps to take, what you have to do to resolve the situation…” (P18, Sp-speaking)*
No additional gain to personal health	*“I think I would want to know more if I were diabetic, if I were healthy, if I were a fit gym rat, I probably wouldn’t concern myself that much with it.” (P12, Eng-speaking)*
Person’s age may influence utility	*“People now in this day in time, my people that I know, my age, they’re enjoying life now to the fullest. They’re not thinking about, ‘What if I have this or that?’” (P10, Eng-speaking)*
**Family health benefits**	*“I think my husband would agree, and my children would agree too. It would be good to know, it would be a warning to them about the risks.” (P23, Sp-speaking)*
**Community health benefits**	*“I feel with the risk scores…you guys could find out more or less on how a disease, how does it affect one race compared to another” (P09, Eng-speaking)*

*Eng*, English; *PRS*, polygenic risk score; *Sp*, Spanish.

**Table 3:** Concerns regarding clinical PRS

Themes/Subthemes	Example Quotes
**Potential for high-risk results to lead to psychological distress**	*“… let us say the person has - comes out with a disease, the person has a disease that they never thought they were going to have, and that can harm them because it can upset them, it can drive them crazy.” (P18, Sp-speaking)*
Concern, worry, and even fear about receiving a high-risk result	*“My concern wouldn’t be about taking the test. I would be concerned about the result of the test. But you have to face it, because it’s a way of seeing one’s life in the future, when you are 60, 70 or 80years old.” (P37, Sp-speaking)*
Anticipating disease onset	*“Well, maybe I’m going to keep thinking that I have those genes and that maybe tomorrow I can develop one of those diseases I mentioned.” (P31, Sp-speaking)*
**Mixed concerns about the accuracy of PRS**	*“… one concern is that it’s still not 100% accurate. So sometimes people might be misunderstood about their health conditions, so that can also influence their lifestyle too, so there’s something I think that we should deal with.” (P04, Eng-speaking)*
Little concern about the accuracy of PRS in diverse populations	*“No. I don’t think there would be any concerns about that… There are no changes, it’s genetics, it’s the beginning, the origin. That’s the way I see it.” (P37, Sp-speaking)*
**Loss of privacy and misuse of genetic and/or health information**	*“… because you don’t know what they can do with the information. I think that scares people more than the test itself.” (P09, Eng-speaking)*

*Eng*, English; *PRS*, polygenic risk score; *Sp*, Spanish.

**Table 4:** Barriers associated with clinical PRS

Themes/Subthemes	Example Quotes
**Access disparities**	*“If the testing is not free, some people who do not have medical insurance may have a problem. If it’s an elderly and you have to move from one location to the next to do the testing, transportation may be a barrier.” (P06, Eng-speaking)*
Financial barriers	*“Maybe they have access to a hospital at a low cost, or they can get help, but they don’t want to skip work because they think they need that money, you know? If they miss one day’s payment, their finances go down.” (P35, Sp-speaking)*
Health insurance coverage	*“Those tests aren’t routine tests and sincerely no one does them and no insurance wants to pay for it, because I know a lot of people that want genetic testing because their mom had ovarian cancer or breast cancer and they don’t want to have them tested.” (P19, Sp-speaking)*
Potential for limited access to clinical PRS on the basis of race and ethnicity or preferred language	*“Sometimes it [genetic testing] may not even be offered to an African-American.” (P03, Eng-speaking)*
**Limited understanding of PRS**	*“I would think, based on how it’s conveyed to the patient, it may be difficult for them to really understand what it is and what the risks are. They may need a little bit of research. They may need to speak to their own physician about what they’re looking at and talk to their peers.” (P27, Eng-speaking)*
Limited health and genomic literacy	*“You have some of those who just don’t understand medical language. You can tell them this until blue in the face, they don’t understand it.” (P05, Eng-speaking)*
Would seek additional information to build understanding	*“…there should be more brochures going around…if you don’t see the information and the doctor doesn’t talk to you about this either, people won’t understand.” (P29, Sp-speaking)*
**Language as a key element to understanding**	*“I can translate from English or Spanish, but my preference would be in Spanish to be able to understand better what they’re talking about, the results, the words you don’t understand, if you don’t get it in Spanish, imagine that, you won’t get them in English, that’s a point that’s important to me.” (P33, Sp-speaking)*
Use of nontechnical lay language is preferred	*“…not all of us understand genetics, it should have terms that are simple and easy to understand.” (P32, Sp-speaking)*
Communicating in patients’ own (preferred) language	*“… the language is very important because sometimes there are people that speak English and they speak a bit of Spanish and they try but they don’t explain things well, so it’s very important, the language the doctor speaks.” (P39, Sp-speaking)*

*Eng*, English; *PRS*, polygenic risk score; *Sp*, Spanish.

## Data Availability

The data that support the findings of this study are available from the corresponding author on request.
