# Supplementary material for: Perspectives of diverse Spanish- and English-speaking patients on the clinical use of polygenic risk scores
Source: Genet Med. Author manuscript; Available in PMC 2023 Apr 1. (PMC10066541; doi:10.1016/j.gim.2022.03.006)
Supplement: Supplementary Material [file NIHMS1869920-supplement-Supplementary_Material.pdf]

Perspectives of diverse Spanish- and English-speaking patients on the clinical use of polygenic risk scores

Sabrina A. Suckiel[1,2], Giovanna T. Braganza[1], Karla López Aguiñiga[1], Jacqueline A. Odgis[1], Katherine E. Bonini, Eimear E. Kenny[1,2,3], Jada G. Hamilton[4,5,6], Noura S. Abul-Husn[1,2,3]

[1]The Institute for Genomic Health, Icahn School of Medicine at Mount Sinai, New York, NY, USA

[2]Department of Medicine, Icahn School of Medicine at Mount Sinai, New York, NY, USA

[3]Department of Genetics and Genomic Sciences, Icahn School of Medicine at Mount Sinai, New York, NY, USA

[4]Department of Psychiatry & Behavioral Sciences, Memorial Sloan Kettering Cancer Center, New York, NY, USA

[5]Department of Medicine, Memorial Sloan Kettering Cancer Center, New York, NY, USA

[6]Weill Cornell Medical College, New York, NY, USA

Supplementary Table 1. Interview guide domains

**Attitudes towards PRS**<br>• General interest<br>• Benefits and harms<br>• Health conditions of most and least interest for learning PRS<br>• Interest modifiers (e.g., disease severity, treatment availability)<br>• Utility and value<br>• Concerns
**Barriers to access, adoption, and use of PRS**<br>• Challenges in obtaining clinical PRS<br>• Challenges in adopting recommendations related to PRS results<br>• Recommendations for addressing cited challenges
**Methods for communicating PRS**<br>• Current method for receiving medical test results<br>• Preferred method for PRS result delivery<br>• Ways to support patient understanding of PRS results<br>• Pre-test and post-test information needs
**Reactions to a hypothetical scenario for hypercholesterolemia PRS; high-risk and not high-risk results**<br>• Interest<br>• Utility and value<br>• Impact on health behaviors and family communication<br>• Reactions to high-risk and not high-risk results<br>• Contextualization of high-risk and not high-risk results

Supplementary Table 2. Interest in clinical PRS themes and subthemes

**Themes /** Subthemes	**Example Quotes**
**Knowledge is power**	*"…knowledge is power in that regard, so I would want to know so that if I ever started to get symptoms or anything and then all of a sudden, something came up on the horizon I would know about it." (P17, Eng-speaking)*
To guide disease prevention and early detection	*"Any disease that can be solved or can be in that blueprint, if there's a way to manage it, then that's the best way to handle it. This way, it doesn't become a medical problem." (P05, Eng-speaking)*
Empowering and boosts self-efficacy	*"When you work hard at finding out what's going on with you health wise, I think that gives you an upper edge in taking control of your healthcare needs as well." (P01, Eng-speaking)*
Improve medical care overall	*"You guys can research and come out with better treatment or a cure if that's what in people lives, because people could feel better moving forward." (P37, Sp-speaking)*
Inform family members	*"About myself, whatever the results, I just want to help my son, because he is the future, and his future is with his children." (P35, Sp-speaking)*
**Interest influenced by family health history**	*"Alzheimer's, I don't have this in my family, that's why I'm not interested in that subject, but regarding the other diseases, I do have relatives that have a certain disease, so, I'm more interested in this than something I don't know about." (P33, Sp-speaking)*
**Trust in medical science**	*"For me to have took the [PRS] test, I would have had to believe in the science first." (P03, Eng-speaking)*
Adoption of PRS would help advance science	*"I think that this type of testing can actually basically change how we see medical science." (P01, Eng-speaking)*

Developing targeted treatments	*“Science should advance at the same pace, so they can treat that genetic deficit, increase it a little bit, and your body would react a lot better.” (P37, Sp-speaking)*
Fear of something new or unfamiliar	*"… no one wants to feel like a guinea pig of trying something new and if there's some defect of some sort, no one wants to be the first to go through the side effects." (P08, Eng-speaking)*
